# Nuclei of *Tsuga canadensis*: Role of Flavanols in Chromatin Organization

**DOI:** 10.3390/ijms12106834

**Published:** 2011-10-14

**Authors:** Walter Feucht, Markus Schmid, Dieter Treutter

**Affiliations:** 1 Unit Fruit Science, Center of Life and Food Sciences Weihenstephan, Technische Universität München, Dürnast 2, D-85354 Freising, Germany; E-Mail: obstbau@wzw.tum.de; 2 Fraunhofer Institute for Process Engineering and Packaging IVV, Giggenhauser Street 35, D-85354 Freising, Germany; E-Mail: markus.schmid@ivv.fraunhofer.de

**Keywords:** nuclei, flavanols, chromatin, cell cycling, meiosis

## Abstract

Needle primordia of *Tsuga canadensis* (hemlock) arising from flank meristems of a shoot apex, form cell lineages consisting of four or eight cells. Within a recently established lineage there is striking uniformity in the pattern of nuclear flavanols. This fact points to an identical transcriptional expression of these flavanols during cell cycling. However two lineages, even if located close together within the same meristem, can be very different in the expression of both cell shape and nuclear flavanol pattern, indicating that epigenetic positional signals are operating in a collective specification of cell lineage development. There is a wide range of nuclear flavanol patterning from a mosaic-like distribution in an activated cell type to a homogenous appearance in silenced cell types. Single cells deriving from lineages are desynchronized because they underlie a signaling network at a higher tissue level which results in stronger epigenetic modifications of their nuclear flavanols. As an extreme case of epigenetic modulation, transient drought conditions caused a drastic reduction of nuclear flavanols. Upon treatment with sucrose or cytokinin, these nuclear flavanols could be fully restored. Analytical determination of the flavanols revealed 3.4 mg/g DW for newly sprouting needles and 19.6 mg/g DW for anthers during meiosis. The roughly 6-fold difference in flavanols is apparently a reflection of the highly diverging organogenetic processes. Collectively, the studies provide strong evidence for combinatorial interplay between cell fate and nuclear flavanols.

## 1. Introduction

A first paper indicating that flavanols (catechins) associate with nuclear chromatin of *Tsuga canadensis* was published by Feucht *et al*. [1]. Later, the studies were extended to a number of coniferous and angiospermous species. By using UV-VIS spectroscopy, it was found that nuclear histones associate with flavanols [2,3]. Final evidence for binding flavanols to nuclei was presented by Mueller-Harvey *et al*. [4]. Continuing histological studies with *Taxus baccata*, it became evident that a special nuclear flavanol pattern is typical for all cells of a distinct cell lineage. Due to fact that each lineage has its own nuclear flavanol state map, it can be deduced that these flavanols are regulated at the transcriptional and translational level [5].

The dynamics of chromatin modification and remodeling are more complex than first envisaged by biologists [6,7]. If flavanols are bound to histones [2,3], then changes in conformation and physiological properties during transcription are virtually inevitable. Low levels of flavanols associated with histone proteins [8] appear to be related to a higher accessible form of euchromatin. Low molecular weight monomeric flavanols as found in the nuclei of conifer species [9] appear to be advantageous in this respect. They associate in a weak and reversible mode with histones. Such a feature allows a highly dynamic and flexible chromatin modification which, according to Kouzarides [10], is of fundamental importance for functional genomes. In contrast, polydentate oligomeric polyphenols are known to bind stably with proteins or even precipitate with them [11].

In recent years, many transcriptional and translational details of histone modification were investigated at the molecular level. However, of similar importance is the large scale nuclear organization in the three-dimensional space. In particular, coniferous species such as *Tsuga canadensis* have been shown to contain nuclear flavanols [2]. The present paper provides new aspects on possible roles of flavanols in *Tsuga canadensis* regarding genome assembly linked with cell cycling, resting nuclei and cell differentiation.

## 2. Material and Methods

### 2.1. Collection Sites and Tissue Sampling

The experiments for the present paper were performed between 2008 and 2010. All work was conducted with four adult trees of *Tsuga canadensis*, between 20 and 30 years old and located in the Botanical Garden in Freising-Weihenstephan. The study trees are located at an elevation of about 550 m. The soil is loamy and deep with a high saturation capacity for water. Shoot growth is vigorous and the needles generally show no signs of injury.

Two further trees of *Tsuga* grow in less fertile soil with little humus layer. Shoot growth is repeatedly moderate to slow because of restricted water use of the canopy in periods with low rainfall. Then, the sun-exposed trees transiently exhibited some incidence of visible injury. Only needles of the current year growth were sampled because even in the one-year old needles the nuclear flavanols fade visibly away, and this is even more apparent in the older needles.

Male cones were sampled from January to late March to investigate the development of the microsporocytes. Sampling during spring from bud break up to late June was performed with newly sprouting needles 2 mm to 15 mm long. From May to July, developing seed cones were sampled to study the young seed wings for nuclear flavanols. In addition, terminal and lateral buds were collected from September until December to study the very beginning of needle development.

Between 50 to 100 nuclei were placed on a microscope slide per one sampling. During the investigation period (2008–2010), between 5000 and 6000 nuclei of *Tsuga canadensis* were studied.

### 2.2. Histochemistry

In principle, only fresh nuclei were investigated by light microscopy because embedding in paraffin caused a significant loss of soluble phenols/flavanols. Moreover, a study of the entire (non-sectioned) nuclei was necessary in order to obtain more insight into the spatio-temporal flavanol distribution. In the case of *Tsuga canadensis* the upper epidermis was carefully removed and the intact mesophyll cells can easily be scraped off with forceps. Among the many conifers investigated in our laboratory, *Tsuga canadensis* in particular is apted to obtain non-ruptured mesophyll cells.

### 2.3. Blue Staining of Flavanols Based on the DMACA Reagent

DMACA staining allows detection of nuclear flavanol spots at a minimum scale of about 1 μm in diameter. Seed wings and primordial meristems of young buds could easily be excised and directly stained without any further manipulation. Staining was performed for 10–20 min with DMACA (1% p-dimethylaminocinnamaldehyde in sulfuric acid, 1.5 M in butanol). Thereafter, the DMACA solution was soaked off, followed by addition of two drops of water which resulted in a rapid change of the tan tissue into a bright blue.

Sulfuric acid of DMACA reagent results in a break of H-bonds of hemicelluloses and pectins of the middle lamella between the neighboring cells. Thus, after staining, the meristematic (shoot tips) and parenchymatic cells of the tissues separated easily from one another when slightly squashed under a microslide. Fortunately, the lineage cells are obviously fastened so strongly to each other that they could be studied in the original cohesive state. Also the cells of the seed wings do not separate from each other. Nevertheless, microscopic examination was not a problem as the seed wings only consist of two cell layers.

DAPI staining (4′, 6-diamidino-2-phenylindole dihydrochloride, Serva) was used for DNA localization under UV light [12]. All colored tissues were examined with a Zeiss microscope, type Axioscop, and the micrographs were made with Agfa color film (CT precisa). Both, cellular and nuclear outlines were measured with Zeiss Axiocam MRC equipment.

### 2.4. Incubation of Tissues

Yellowed needles (cells) were treated with cytokinins (cytokinin benzylaminopurin (BA) 1 μM–10 μM) and sucrose (5–10%) for 54–60 h. As an alternative, intact short shoots 2 cm in length were placed in small tubes with solutions of cytokinins or sucrose (concentrations as mentioned above). Both compounds were transported via the xylem into the young needles, shoot tips or buds. During a long dry period in 2007 the experiments were repeated 6 times with tissue samples each containing 10 needles. Per needle roughly 150 nuclei were checked. About 50 seed wings sampled in May were incubated for 50 h in watery IAA (indole acetic acid) solutions at 28 μM. The development of nuclei and particularly of nucleoli was then checked by microscopy.

### 2.5. Densitometric Studies

**Figure 1:**
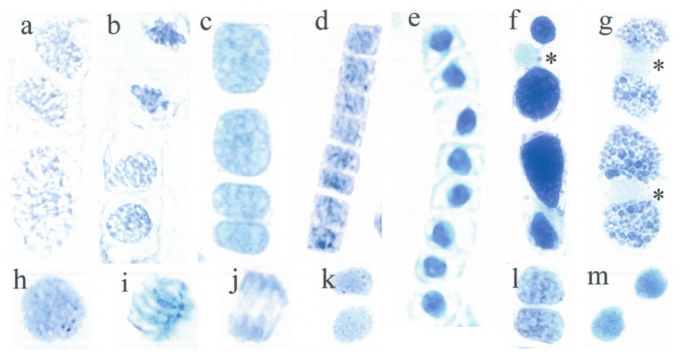

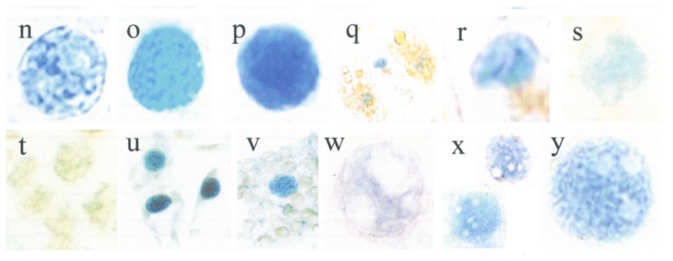
Nuclei of *Tsuga canadensis* with different nuclear flavanol patterns. The nuclear size in images n to w is between 7–8 μm in diameter. In all other images the nuclear size is given. (**a**) Lineage with 4 cells: An early telophase (above) and a prophase (below, 22 μm in length) with an activated flavanol pattern; (**b**) Lineage with 4 cells: An early telophase (above) and two newly formed interphase nuclei (below, rounded nuclei 7 μm); (**c**) Lineage with 4 cells, two rounded interphase nuclei and two flattened nuclei just after cytokinesis. The nuclear flavanol pattern is a moderate mosaic, indicating a moderate activity (upper nucleus, 8 μm in length); (**d**) Lineage with 8 cells. During the endphase of cell cycling, the nuclear flavanol pattern is characterized by mosaic-like intermingling of euchromatin and heterochromatin (uppermost nucleus 8 μm in diameter); (**e**) Lineage with 8 cells. After exit from cell cycling the silenced nuclear pattern is revealed by dense, diffuse blue flavanols (uppermost nucleus 6 μm in diameter); (**f**) Two vacuolated cells with large vacuoles and very pale, evenly diffuse nuclei indicating a silenced nuclear flavanol pattern (upper nucleus 7 μm in diameter); (**g**) Two vacuolated cells with multiple flavanol inclusions. Very pale evenly diffuse nuclei indicate a silenced nuclear flavanol pattern (upper nucleus 8 μm in diameter); (**h**) Prophase nucleus with a fine-granulated smooth flavanol pattern (9 μm in diameter); (**i**) Metaphase with pale diffuse chromosomes and some denser blue regions along the equatorial plate; (**j**) Telophase with diffuse appearance and diffuse flavanols lagging behind the chromosomes; (**k**) Two daughter nuclei with very slight differences regarding the fine-granulated nuclear flavanol patterns (lower nucleus 7 μm in diameter); (**l**) Two daughter nuclei with almost identical nuclear pattern of intermingled euchromatin and heterochromatin (upper nucleus 8 μm in diameter); (**m**) Two daughter nuclei with diffuse but slightly different nuclear flavanol patterns (both nuclei 8 μm); (**n**) Single nucleus with a highly activated, mosaic-type flavanol pattern; (**o**) Single nucleus with a nearly diffuse moderate blue flavanol pattern covered with short streaks of chromosomal sectors; (**p**) The heavy blue and diffuse staining nucleus is functionally highly repressed; (**q**) Drought stress imposes accumulation of “yellow” flavonoids in the cytoplasm. The nuclear flavanols fade away. As a control, midway between both cells, a single nucleus with moderate flavanol affinity; (**r)** Magnification of the single nucleus shown in q; (**s**) Greenish staining nucleus within the yellow cytoplasm, rich in flavonoids; (**t**) After a ten day drought period all nuclei of a needle lost the nuclear flavanols; (**u**) Incubation in cytokinin resulted in recovery of the nuclear flavanols. Different staining intensity of the nuclei points to an individual import facility for flavanols; (**v**) Incubation in sucrose resulted in recovery of nuclear flavanols; (**w**) Nucleus from a seed wing treated with IAA was induced to develop three large nucleoli. (IAA produces a reddish tint with the DMACA reagent); (**x**) Nuclei of meiotic pollen mother cells with varying expression of nucleoli (nuclei 9 μm in diameter); (**y**) Magnified nucleus of pollen mother cells with three nucleoli and a highly activated mosaic-like flavanol pattern (nucleus 9 μm in diameter).

The blue colored micrographs of the nuclei were scanned using Nicon equipment (Cool Scan IV E D). Both the density and subnuclear distribution of flavanols were imaged at the nuclear level. A very dense blue stained nucleus ((Figure 1(p)), Table 1) was used as a maximal internal staining standard with an absorbance of 100% (A 640) towards zero (0%) after heat stress ((Figure 1(t)), Table 1). All results were normalized to this internal standard.

The use of a spectral photometer CM-700d (Konica Minolta Sensing, Nieuwegein, The Netherlands) was absolutely necessary to determine the degree of the mosaic-like intranuclear flavanol expression as a means of the nuclear loosening and activity. The entire complex of the flavanol-free white colored interchromosomal space and blue staining nuclear flavanol spots, being 1 μm or less in size, can be reliably measured in this way.

The scanned micrographs of the nuclei or vacuoles (Figure 1) were measured at eight different positions. Then, the arithmetric average values and standard deviation (SD) were calculated. The Anderson-Darling test, a statistical tool for detecting departures from normality, was used to prove whether a normal distribution adequately describes the sets of data [13]. In all cases the hypothesis of normality was validated at a 5% level test.

### 2.6. Analytical Determination of Flavonoids by HPLC

Phenolic compounds were separated on a column (250 × 4 mm I.D.) prepacked with Hypersil ODS (3 μm particle size). The HPLC system consisted of an autosampler (Gilson-Abimed Model 231), two pumps (Kontron Model 422) and a diode array detector (Bio Tek Kontron 540). For post column derivatization, an additional analytical HPLC pump (Gynkotek Model 300 C) and a VIS detector 432 were used. Flavanols and flavones were detected at 280 nm. Flavanols were stained with 1% DMACA in 1.5 N H2SO4 in methanol to produce a colored complex with a peak absorbance at 640 nm.

Stepwise gradients were applied using mixtures of solvent A (formic acid, in water) and solvent B (methanol) from 95:5 (v/v) to 10:90 (v/v) with a flow rate of 0.5 mL min-1 [14]. The gradient used was: 0–5 min, isocratic, 5% B in A; 5–15 min, 5–10% B in A; 15–30 min. isocratic, 10% B in A; 30–50 min, 10–5% B in A; 50–70 min, isocratic, 15% B in A; 70–85 min, 15–20% B in A; 85–95 min, isocratic, 20% B in A; 95–110 min, 20–25% B in A; 110–140 min, 25–30% B in A; 140–160 min, 30–40% B in A; 160–175 min, 40–50% B in A; and 175–190 min, 50–95% B in A.

### 2.7. Peak Identification

Flavonoid peaks were identified based on UV absorbance, chromatographic behavior on HPLC and in the case of flavanols the development of a blue color following post column derivatization and TLC (thin layer chromatography). Catechin, epicatechin and epigallocatechin were used as standards (Roth, Karlsruhe) and oligomers (proanthocyanidins) were identified according to their chromatographic behavior [14]. Identification of flavonols was performed by comparing their chromatographic behavior on HPLC and TLC and their UV spectrum with those of standards (quercetin, myricetin, apigenin, kaempferol, luteolin). Cellulose plates (Merck) were used for two-dimensional separation (TLC) of flavanols. The solvents were: first direction, n-butanol: acetic acid: water (BAW, 4:1:2.2 v/v); second direction 10% formic acid. Flavanol spots were visualized by spraying with DMACA reagent. For quantification by HPLC of the monomeric catechins the response factors of the corresponding authentic standards were used. Their oligomers, the proanthocyanidins were estimated as procyanidin B2 which was previously isolated in our lab. Hydroxycinnamic acids and flavonols were calculated as chlorogenic acid and rutin, respectively.

## 3. Results

### 3.1. Short Cell Lineages

Cell lineages mainly develop in the very early growing season. Most lineages of *Tsuga canadensis* consist of four cells (Figure 1(a–c)). The three lineages shown here (Figure 1) were at different development stages. There was a wide range of chromatin density (absorbance in %) and intranuclear variation coefficients. Both the flavanol density and distribution were quantified on the basis of densitometry (Table 1).

The first example (Figure 1(a)) provides structural details of a prophase and telophase on the way to establishing four cells. The mitotic prophase spread just before entering into the metaphase ((Figure 1(a)), below) was notably expanded in size. The telophase (Figure 1(a)), above) showed a very regular and fine grained mosaic pattern of the chromosomes to achieve compaction of the newly forming haploids.

In (Figure 1(b)), lineage cell formation was more advanced but likewise not fully synchronized. Most conspicuous were the rather compacted mid-telophase aggregations with densely arranged dark blue chromatin flavanols. The formerly stretched anaphase chromosomes were now replaced by thickened short streaks. Regarding the two circular progeny nuclei (Figure 1(b)), cytokinesis was already completed and the diameter of the newly developed interphase nuclei (7 μm in diameter) was already in the usual range.

Mitotically most advanced in development were the four interphase nuclei which, however, still had not reached full developmental synchrony (Figure 1(c)). The four daughter nuclei stained a fairly blue but rather diffuse mosaic pattern, with the interchromosomal areas being of a pale blue shade.

The nearly rounded pair of daughter nuclei were organized absolutely similarly in size (8 μm in length), shape and nuclear flavanols. The two lower progeny nuclei (Figure 1(c)), with a short delay in forming a rounded shape, had just completed cytokinesis. Despite the slightly asynchronous timing of the cell cycles, there was outstanding uniform expression of the nuclear flavanol patterns of all four nuclei. The clonal character of the four haploid daughter nuclei realized perfectly.

### 3.2. Long Cell Lineages

Sometimes, eight longitudinally arranged cells (Figure 1(d,e)) arise from an initial meristem cell by three sequental divisions. Both distinctly diverging lineages were positioned in the same needle primordium, only about 100 μm away from each other. Such a distance is obviously enough to evoke different position-dependent signals. The first lineage with the quadrangular cells and nuclei was actively running through the final stage of cell cycling (Figure 1(d)) whereas in the second lineage, consisting of rounded nuclei, a block of cell division is linked with enforced cell expansion (Figure 1(e)). The activated nuclei are typically characterized by a rather dense but structurally typical mosaic flavanol pattern (Figure 1(d)) which is very different from the compacted, evenly diffuse pattern of the second lineage (Figure 1(e)). Thus, the mean cell/nucleus ratio amounted to 1.3 for the activated lineage and 3.3 for the silenced one (Table 2).

The darkest heterochromatic blobs of the mosaic-like nuclei might be qualified as overexpressed in flavanols. Even the thin band of cytoplasm around the nuclei, being normally without flavanols, showed a quite unusual tint of blue stained flavanols which can be interpreted as evidence of abundant flavanol synthesis by cytoplasmic ER. In this context, the lowermost mosaic-like cell pattern showed a rather high absorbance value and a high standard variation (Table 3). Such a deviation is imposed by epigenetic factors which were obviously capable of producing first symptoms of chromatin modification overriding the genetically controlled cell fate. Quite different from the lowermost cell number 8 was number 1 from this lineage (Figure 1(d)) which showed a low absorbance value combined with a very low standard deviation.

The human eye, however, sees a more fine-tuned impression of nuclear flavanol patterns. Thus, the activated lineage (Figure 1(d)) exhibited slight differences in the nuclear flavanol pattern among the four daughter cell pairs (numbers 1 + 2, 3 + 4, 5 + 6, 7 + 8, Table 2).

### 3.3. Vacuolate Cells with a Silenced Nuclear Flavanol Pattern

During the early period of seed wing development most cells were more or less isodiametric. Lateron, the cells increase in size and often files of elongating cells with very large vacuoles were formed (Figure 1(f)). By that time, abundant amounts of flavanols were accumulated (Figure 1(f)). However, there was a dramatic difference between vacuolar and staining intensity of the resting nuclei. The homogenous flavanols of the nucleus (*) were of a pale, even blue color, and the standard deviation was only 1%. (The second pale nucleus of the lower cell is partially covered by both dark blue staining vacuoles).

A drastic modification of the vacuolar morphology is seen in (Figure 1(g)). Numerous mini-inclusions, between 0.5 to 2 μm in diameter, were densely assembled within one large vacuole. The nuclei (*) with only a bluish tinge were found to be in close contact with the storage phenols.

### 3.4. Daughter Cells Display a Nearly Similar Nuclear Flavanol Pattern

In young needles, certain parenchyma cells located outside the meristems can sometimes occasionally re-enter into cell cycling. Both derivative daughter cells apparently produce almost equal amounts of nuclear flavanols.

In the prophase (Figure 1(h)), the slightly enlarged nucleus (9 μm in diameter) has typically lost the silent state. Instead, there was a characteristic fine-granular spotting of chromatin with a group of only three closely located, punctuate knobs of heterochromatin (<1 μm in diameter). In principle, prophase nuclei undergo a characteristic chromatin reorganization and spiralization of chromosomes.

Metaphase chromosomes (Figure 1(i)) are compacted and structurally diffuse in their flavanol structures. The long-armed chromosomes (up to 8 μm) are partially very diffuse. There were a few concrete dark blobs located along the metaphase plate. They might be defined as heterochromatic aggregations. Perhaps, cyclin-dependent kinases are inhibited by a locus-specific compaction of chromatin. All in all, the variability in flavanol density of this metaphase spread was relatively high.

In the anaphase-telophase (Figure 1(j)) there was a pronounced diffuseness of flavanols all over the chromosomes. Some of the diffuse blue structures lagging behind the bulk of chromosomes are probably freely moving flavanols, or transported by vesicles.

After cytogenesis, the genetically equivalent pairs of progeny nuclei might gradually be exposed to slight deviation from the clonal character. For example, one of the two daughter cells (Figure 1(k)) was slightly denser in both euchromatin and in the number of fine-structured mini-blobs. Likewise, the following two progeny nuclei differed from one another by the intensity of blue heterochromatic patches (Figure 1(l)). Finally, a dense, diffuse nuclear flavanol pattern was common for both daughter nuclei (Figure 1(m)), although a slight difference was evident.

Clearly, each pair of progeny nuclei can be distinguished from the other pairs by their shape, size and flavanol pattern (Figure 1(k,m)). Such differences are epigenetically driven by local and temporal oscillations in transcription. Sometimes, in still young needles certain parenchyma cells located outside the meristems occasionally can re-enter into cell cycling. Both derivative daughter cells apparently produce almost equal amounts of nuclear flavanols.

To begin with prophase (Figure 1(h)), the slightly enlarged nucleus (9 μm in diameter) has typically lost the silent state. Instead, there was a characteristic fine-granular spotting of chromatin with a group of only three closely located, punctuate knobs of heterochromatin (<1 μm in diameter). In principle, prophase nuclei undergo a characteristic chromatin reorganization and spiralization of chromosomes.

Metaphase chromosomes (Figure 1(i)) are compacted and structurally diffuse in their flavanol structures. The long-armed chromosomes (up to 8 μm) are partially very diffuse. There were few concrete dark blobs located along the metaphase plate. They might be defined as heterochromatic aggregations. Perhaps, cyclin-dependent kinases are inhibited by a locus-specific compaction of chromatin. All in all, the variability in flavanol density of this metaphase spread was relatively high.

At anaphase-telophase (Figure 1(j)) there was a pronounced diffuseness of flavanols all over the chromosomes. Some of the diffuse blue structures lagging behind the bulk of chromosomes are probably freely moving flavanols.

After cytogenesis the genetically equivalent pairs of progeny nuclei might gradually be exposed to slight deviation from the clonal character. For example, one of the two daughter cells (Figure 1(k)) was slightly denser in both euchromatin and in the number of fine-structured mini-blobs. Likewise, the following two progeny nuclei differed from one another by the intensity of blue heterochromatic patches (Figure 1(l)). Finally, a dense, diffuse nuclear flavanol pattern was common for both daughter nuclei (Figure 1(m)), although a slight difference was evident.

Clearly, each pair of progeny nuclei can be distinguished from the other pairs by their shape, size and flavanol pattern (Figure 1(k,m)). Such differences are epigenetically driven by local and temporal oscillations in transcription.

### 3.5. Variable Nuclear Flavanol Pattern of Fully Differentiated Single Cells

As shoot development proceeds, the mature cells might undergo different states of specialization, and nuclear flavanol patterning follows this diversification. Three extreme examples of different nuclear flavanol patterning are shown (Figure 1(n,o,p)). The pronounced mosaic-type nucleus (Figure 1(n)) is to be qualified as a mottled pattern of open-structured euchromatin intermingled with compacted heterochromatin. Thus, the absorbance value was moderate and the intranuclear variation was relatively high. In addition, even colorless domains were recognizable. An interphase nucleus with prominent patches of heterochromatin along with very pale domains of euchromatin and colorless interchromosomal spaces is considered to be in a transcriptionally active state.

Another interphase nucleus with a more homogenous, silenced developmental stage (Figure 1(o)) stained a paler, diffuse blue-greenish tint with several slightly blue, short threads distributed throughout the nucleus. As illustrated in (Figure 1(p)), exceptionally abundant flavanols with an overall extreme blue staining reaction distributed diffusely throughout the nucleus represent a maximally silenced chromatin state. This view is supported by its position in a mature seed wing. The relative absorbance of this nucleus was estimated as maximal (100%) when applying densitometry, but the SD value was reduced to zero (Table 1).

With advancing development the needle cells were exposed to repeated stress events. A few days of intense light and drought exposure, particularly at the southern canopy site, resulted in chlorotic areas which were initially 3 to 4 mm long. This was most pronounced in the upper epidermis but cytoplasm and variable-sized plastids of mesophyll cells likewise turned yellow in color (Figure 1(q)). Their nuclei indicated a loss of flavanols which resulted in a greenish tint as a mixture of blue and yellow (Figure 1(q)). At mid-distance between two yellow colored cells was one cell nearly devoid of yellow components with the nucleus showing a bright blue (Figure 1(q)). If magnified, this nucleus indicated a quite diffuse, wavy flavanol pattern, with few hazy, darker blue areas (Figure 1(r)). These symptoms became more pronounced as the radiation stress progresses, giving rise to a greenish-blue diffuse and much less densely stained nucleus (Figure 1(r,s)). In both nuclei the crucial point was the loss of any structures within the nuclei which is particularly evident in the case of (Figure 1(s)) by a very low absorption and SD values. The ultimate fate was a complete loss of the nuclear flavanols (Figure 1(t)).

Simple application of cytokinin (1–10 μM) resulted in a drastic return of the nuclear flavanols, often with a different degree of blue staining, pointing to an individual import capacity of the nuclei (Figure 1(u)). Therefore, the corresponding mean absorbance values of the three nuclei were rather high, reaching 88.4% (Table 1). Return of blue nuclei also occurred when using 10% sucrose as an incubation medium (Figure 1(v)).

### 3.6. Nucleoli of Somatic Cells of Tsuga canadensis Are not Recognizable by DMACA Staining

Active nucleoli are the sites of pre-ribosomal gene transcription, rRNA synthesis and packaging of ribosome subunits. Normally, in all coniferous species studied up until now the nucleoli of needles and shoots did not stain with the DMACA reagent. Instead, they appeared as a white hole in the blue nucleoplasm. However, this does not apply to the nucleoli of *Tsuga canadensis*. Moreover, DAPI staining (yellow fluorescence of flavonoids) revealed normal frequencies of nucleoli in *Tsuga canadensis*. These nucleoli were mainly small, about 2 μm in diameter, and were generally not sharply confined. It can be concluded from these experiments that free flavanols are likely located in the nucleoli.

When nuclei from *Tsuga* seed wings were treated for two days with auxin (IAA at 10 mg/L), the use of DMACA reagent revealed relatively large white nucleoli in about 15% to 20% of the nuclei (Figure 1(w)). The reddish shade of the nucleus is due to added IAA because indoles react this way with DMACA. (Regarding the relative absorbance, the very large nucleoli were not included in the measurements, Table 1, (Figure 1(w))).

### 3.7. Nucleoli of Sporogenic Cells Are Detectable by DMACA Staining

Surprisingly, the use of DMACA reagent to investigate male cones during microspore development revealed a high frequency of nucleoli in pollen mother cells (77%) shortly before tetrads are formed. However, most of the pollen mother cells (not shown) were unique insofar as the cytoplasm surrounding the nuclei was heavily stained for flavanols. Thus, the nucleus itself generally is not recognizable. At the start of tetrad formation the blue color of the cytoplasm starts to slowly fade away. A certain percentage of the tetrads (22%) contained nucleoli. Two tetrad nuclei, already free of a blue cytoplasm, are shown (Figure 1(x,y)). Up to six nucleoli were found, the larger ones reaching about 2 μm in diameter. As illustrated (Figure 1(x)), the upper nucleus showed a slightly more pronounced mosaic-like flavanol pattern compared to the lower one. To show the actual flavanol configuration of a tetrad nucleus more precisely, a magnified picture shows a fine granulated uniform mosaic-type expression (Figure 1(y)) pointing to a transcriptionally active genome configuration. As a general rule, the cytoplasmic flavanols fade completely away in the late tetrad stage.

### 3.8. Flavanols and Other Phenolic Compounds during Development of Microspores and Young Needles

The pollen mother cells were relatively large (>15 μm in diameter) and at early stages of development both nucleus and cytoplasm mostly stained a dark blue for flavanols. All other cells of the anthers from tapetum to epidermis showed rather small cells which seldom contain vacuoles. The analytical data (Table 4) comprise the entire anthers, so a very limited number of vacuoles filled with flavanols are included in the analytical data.

Based on HPLC analysis, the monomeric flavanols catechin and epicatechin were predominant making up 8.6 and 7.1 mg/g DW each. Epigallocatechin was much reduced in concentration, reaching only 0.6 mg/g DW. In addition, 3.3 mg/g DW unidentified flavanols were present, probably consisting of dimeric molecules and perhaps traces of oligomeric flavanols. Other phenolic compounds such as hydroxycinnamic acids and flavonols were likewise present.

Emerging needles, 2–8 mm long, were also sampled as they represent optimal growth conditions. However, even these young leaves revealed a number of enlarged cells with flavanol-storing vacuoles. Thus far, the flavanols of the leaflets shown in Table 4 refer not only to nuclei.

The needle flavanols mainly consisted of the fundamental structural units catechin and epicatechin (Table 4), whereas epigallocatechin was present at rather low levels. The monomeric flavanols in total reached 2.5 mg/g DW. In addition, a few dimeric and probably a few oligomeric proanthocyanidins, amounting up to 0.9 mg/g DW, should not be overlooked. Notably, the hydroxycinnamic acids were very high, accounting for 161 mg/g DW. Regarding the flavonols, the concentrations were extremely low (0.3 mg/g DW) compared to the hydroxycinnamic acids (Table 4).

### 3.9. Densitometric Scanning and Expression of Chromatin Activation (Table 1)

Using densitometric scanning at A640 nm it is possible to define more precisely the terms pale, moderate or dark blue by using relative absorption values. It is an important aspect that roughly 5 fold differences between the lowest (pale blue) and highest (dark blue) absorption exist in nuclei (Table 1). The biological relevance of such a large range in flavanol binding properties points to a high degree of adaptive flexibility to a given situation. Much larger still is the 10 to 100 fold difference of intranuclear flavanol variation (SD), for example from 0.2 (Figure 1(g)) to 20.8 ((Figure 1(d)), Table 1). Gene suppression or activation can be described more precisely approaching the intranuclear SD values. The degree of mosaic patterning highlights the particular importance of both non-histone proteins and histones in inducing a high nuclear activity. In contrast, pronounced diffuseness of the flavanols and loss of any mottling of flavanols hints at down-regulated silencing of signaling pathways and nuclear activity. As shown in (Figure 1(f,g,s)), it is clearly apparent that the immense complexity of the multiple nuclear regulatory networks cannot be determined by the flavanolic content alone.

## 4. Discussion

### 4.1. Highly Mitotic Cells Possess a Mosaic-Like Nuclear Flavanol Pattern

Stem cells produce founder cells which then form cell lineages in the lateral meristems of the apex [15]. Such a lineage is the result of rapid cell cycling with kinesin proteins acting as crucial components of the mitotic cell machinery [16]. In principle, in a young cell lineage there is exists a strong cell to cell adhesion providing highly effective intercellular molecular networks. This effect is based on a physical contact mediated by adhesion molecules and by well-functioning plasmodesmata. The latter allow a mutual interplay of intercellular ribonucleoprotein signaling [17].

In meristems of *Tsuga canadensis* there were newly developing lineages with four cells which form quite different patterns of nuclear flavanols (Figure 1(a–c)). The nuclear structures were highly loosened so that the chromosomes are spatially localized in rather distant specific territories ((Figure 1(a,b)), Table 1). As a particular aspect, the interchromatin areas were devoid of flavanols (Figure 1(a,b)). Active multiprotein complexes and numerous non-histone effector proteins are thought to be localized in these chromosome-free areas [18]. In such an activated environment the chromatin histones are hyperacetylated and transcriptionally upregulated [10]. Such a situation corresponds to Tsuga canadensis (Figure 1(a–d)) in that a loose assembly of histone-DNA structures keeps the nucleosomes weakened to undergo significant chromatin modifications related with mitotic cell cycling.

As can be seen in the four-celled lineage (Figure 1(c)), the darker blue streaks were embedded in a pale blue environment and the question arises posed whether there might be freely moving flavanols (a connection is not necessarily the case) or scarce, pale staining flavanols bound to histones. As pointed out by Misteli [19], chromatin can diffuse freely through the nucleoplasm because subnuclear domains are not delineated by membranes. The significant differences in the nuclear flavanol pattern are mainly caused by changes of the interchromosomal space (Figure 1(a–c)). Freely moving histones were found during cell cycling, particularly during the S-phase when DNA replicates [20]. In this context it should be emphasized that interchromosomal matrix structures readily play a major role in essential nuclear functions [21].

### 4.2. Different Flavanol Expression of Two Cell Lineages Located Close Together

Most impressive were two eight-celled lineages which arose from three rounds of division and dramatically differ in the large scale chromatin organization, although their spatial distance from one another in the needle meristem was only about 150 μm ((Figure 1(d,e)), Tables 1, 2, 3). The small-celled mitotic lineage revealed a mosaic-like appearance of the nuclei (Figure 1(d)). This contrasted with the longer cell lineage whose nuclei had the appearance of compact blue stained balls (Figure 1(e)). Within a given cell lineage or a meristem a tight control of cell division and morphogenetic patterning of new cells is mainly regulated by diverse signals and hormonal impulses deriving from neighboring cells [22]. Additionally, special functional short range signals coordinate transcriptional activities [23,24]. Scholten *et al*. [25] emphasize that a controlled genomic condensation and decondensation during transcriptional activity is the rule. This feature is consistent with the striking mosaic-like appearance of the nuclear flavanols in the mitotic lineage cells (Figure 1(d)). Chromatin is a dynamic continuum regulated by epigenetic modifications of the nucleosomal structures [26]. Strong repression of distinct sets of genes is a prerequisite for transcriptional expression of another set of genes [27,28]. Massive upregulation of flavonoid genes mostly results in downregulation of other subsets of genes [29].

Figure 1d shows a predominance of dense blue heterochromatin interruptured by nearly colorless domains of pale euchromatin. Because of the special local arrangement, Carmo-Fonseca [30] prefers to denominate the heterochromatins as compartments. The fact that flavanols are involved in loose and dense modification of chromatin offers a new conceptual insight into the multi-facetted transcriptional networking.

Considering the high sink demand for flavanols in the enlarged, newly developing nuclei ((Figure 1(d)), Tables 2, 3), the biosynthetic flavanol machinery of cytoplasmic ER has to run on high speed. Indeed, the relatively small vacuole-free cytoplasmic rims stained a quite unusual blue, not common for cells of *Tsuga canadensis*. Rough ER is distributed throughout the cytoplasm but in part directly aligned to the nuclear envelope which then is most licenced to supply the nucleus with flavanols, probably by way of vesicle-like prevacuolar compartments.

The second cell lineage showing an absolutely even blue nuclear flavanol pattern (Figure 1(e)) advocates overall enforced gene repression. These compacted nuclei only measured nearly half the perimeter of the active ones. Such a compression is causally linked with repression of transcription keeping genes inactive [31]. Any access of signals, hormones, proteins and enzymes to DNA is blocked upon dense compaction of chromatin [32].

**Figure 2:**
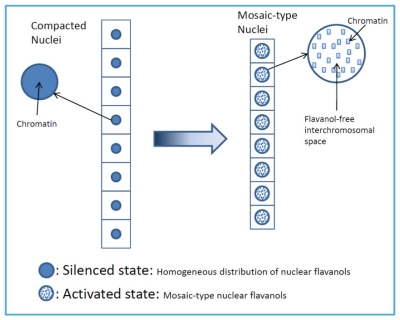
Differently packed chromatins (corresponding to the two eight-celled lineages from colored (Figure 1(d,e))).

Figure 2 gives a schematic representation of the principal nuclear flavanol patterns of silenced and activated cells.

### 4.3. Daughter Cells and Beyond: The Nuclear Flavanol Pattern Linked with Progressing Differentiation

**Figure 3:**
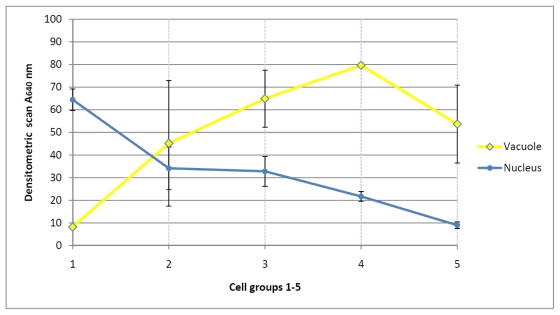
Densitometric scanning of flavanols (A640 nm) of nuclei and vacuoles. Mean values and SD of five groups with different sizes and densities of nuclei and vacuoles.

Overloading of a mature daughter cell with abundant vacuolar flavanols likewise results in evenly structured nuclear flavanols, typical for silencing (Figure 1(f,g)). However, the densities of the nuclear flavanols range over a rather low level (Figure 1(f,g)). This is best exemplified in seed wings showing deposition of abundant vacuolar flavanols to be linked with nuclei being poor in flavanols (Figure 3). The vacuolar flavanols of the seed wings begin to age and oxidize in late autumn. Obviously, also the nuclei go through a state of pre-senescence so that the flavanols fade slowly away.

Cell cycling outside meristematic domains but in elongating tissues occurred occasionally within more or less differentiated cell groups of *Tsuga canadensis*. Within these cell clusters distinct single cells were specified to re-enter into a single cell cycle event, evoked by a very local spatio-temporal expression of favorable levels of auxin and cytokinin [33,34]. Shortly after cytokinesis those daughter cells of *Tsuga canadensis* showed apparently similar transcriptional signals inherited from their mother cell regarding both flavanol concentration and flavanol pattern.

Regarding the prophase (Figure 1(h)) there were three prominent dark blue blobs of heterochromatin. These compartmentalized subsystems remain condensed also during the cell cycle, and result from histone hypoacetylation [28]. The metaphase chromosomes (Figure 1(i)) showed some condensed heterochromatic domains. If located along the metaphase plate, they might be linked with cohesion of daughter chromosomes near centromers [35].

The two haploid telophase aggregations were, as expected theoretically, equal in the packaging intensity of the flavanols (Figure 1(j)). After cytokinesis was completed, the newly established daughter nuclei were at first sight found to be identical in shape, size and flavanol organization ((Figure 1(k,l,m)), Table 2). However, on more precise examination, minute differences in flavanol density became apparent (Figure 1(k,m)). Evidently, modifying epigenetic mechanisms such as acetylation, loosening of DNA-histone interactions [36] or methylation came into play soon after the daughter nuclei were re-established. A combined role of histone and non-histone proteins is thought to be causally involved in packaging of DNA [28]. The question arises as to what extent flavanols might contribute to these mutual mechanisms.

Continuing a cell’s life span beyond the state of daughter cells means a prolonged influence of epigenetic interaction networks with a distinct specialization towards a more individual development. With progressing age of cells a major impact of external signals on nuclear flavanol expression takes place since the plant needs a steady adjustment to environmental changes (Figure 1(n–s)). Then, a high degree of transcription, that is both packaging and opening of nucleosome chromatins, might pass on to single cells (Figure 1(n)). Evidently, euchromatic gene activation and heterochromatic gene silencing were combined in this nucleus.

**Figure 4:**
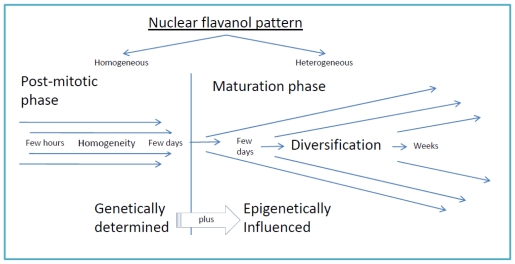
Similar nuclear pattern of post-mitotic daughter cells and diversification during cellular differentiation.

However, there were also nuclei with a rather uniform over-expression of flavanols (Figure 1(o)). This might cause, at least transiently, a block of notable activity and transcription. A further example (Figure 1(p)) shows a lack of any spatial flavanol patterning in a dark blue nucleus of mature seed wing. As this nucleus was the only one throughout the entire seed wing, genetic defects should be taken in consideration. Mitotic defects can produce chromosomal hypercondensation [16]. A schematic representation of the changing nuclear flavanol pattern during cell development and diversification is shown in Figure 4. Just after cytokinesis, few hours up to few days, the nuclear flavanol pattern is rather homogenous because the daughter cells behave like clones. Later on, developmental diversification during a time span of a few days or weeks, the epigenetic modifications become much more pronounced. Therefore, both cell shape and cell size change in concert with the nuclear flavanol pattern which then shows distinct modifications from diffuse to mosaic or from pale blue to dark blue.

### 4.4. Epigenetic Stress, Loss of Nuclear Flavanols and Recovery by Sucrose and Cytokinin

Intense UV-light is in principle dangerous for plant nuclei [37]. Regarding needles of *Tsuga canadensis* minor changes from dark green to light green needle sectors could be found frequently and the blue stained nuclear flavanols are smooth in appearance which lateron change to a greenish stain. (This is due to a mixture of yellow flavonoids with the blue flavanols as in (Figure 1(s)). As reviewed by Treutter [38], flavonoids and flavanols play a multifaceted role in many plant species in responding to permanently changing environmental conditions. If the nuclei of *Tsuga canadensis* acquired a very pale and diffuse flavanol pattern without any dots, stipples or prominent chromatin blobs, then a type of senescence is apparently achieved.

Experimental addition of cytokinin to the heavily stressed nuclei rapidly restored the normal blue flavanol reaction (Figure 1(v)). Numerous papers indicate senescence retarding properties of naturally occurring cytokinins [39].

Also nutritional effects and hormonal gradients may function in this way. Sucrose, often attracted by cytokinins, also restored the loss of nuclear flavanols of *Tsuga canadensis* during transient drought periods. In a number of cherry cultivars (*Prunus avium*), the flavonoid naringenin 7-glucoside was more than doubled by treatment with benzyladenine [40]. (Rosacean fruit species do not develop nuclear flavanols). Cytokinin signal transduction is encoded by a gene family and it is well known that this hormone might improve the nutritional status of plants or special plant tissues respectively [41,42]. More precisely, cytokinin has a direct effect on transcriptional activation of rRNA gene promoters [43].

The increase of PAL activity in response to sucrose has been well known for a long time [44]. Sugar-regulated gene expression coordinates development of plants with availability of nutrients [45]. Chromatin is considered to be the natural substrate for genome-based transactions as compared with naked DNA [46]. The introduction of flavanols into chromatin biology provides novel fundamental functions for these low molecular weight phenols.

### 4.5. Nucleoli of Somatic and Meiotic Cells

The nucleoli of somatic cells from *Tsuga canadensis* was scarcely found during the past twelve years of investigating this species by DMACA staining. According to Nitsch [47], treatment of nuclei from tobacco with auxin (1 mg/L) resulted in enforced expression of nucleoli. This finding was confirmed by external application of auxin to seed wings of *Tsuga canadensis*. Evidently, the growth hormone was capable of significantly enlarging and activating the nucleoli. It might be argued that the nucleoli are occupied by freely moving catechins. In addition to these aspects, Burger and Mueller [48] claimed that in *Vicia faba,* nucleolar chromatin is synthesized along with endomitosis and development of nucleolar organizer regions (NORs).

Rather unexpectedly, in contrast to somatic nuclei, it was easy to recognize a high frequency of nucleoli in meiotic nuclei of male cones. They appeared as unstained subcompartments after applying DMACA. Up to six nucleoli could be observed in tetrads which were diminished just before transit to the uninuclear stage of early microspores. The nucleolar area correlates with the activities of rRNA genes [49,50]. Meiosis involves the interaction of many genes, which is reflected by high transcriptional activities. In young pollen mother cells of wheat and rye, multiple nucleoli could be found in the peripheral regions of the nuclei which later fuse to a smaller number [51,52].

Meiosis itself is a complicated process with highly expressed transcriptional activities involving synthesis of proteins and pre-ribosomal structures. DNA lesions during S-phase progression, homologous pairing, and recombination processes create the danger of meiotic irregularities [53]. Too high polymerase activity during DNA duplication would be disastrous. Indeed, flavanols were successful in reducing the polymerase activity of mammalian DNA [54]. Compared to the somatic needle cells, the very high flavanol loading of the meiocytes, up to 6-fold, is probably involved in stabilizing the genome integrity during this critical developmental phase. Stabilization means that a subset of genes is suppressed to a silenced conformation. The mosaic-type flavanol pattern of the tetrad nuclei reveals both silenced and activated genetic domains.

It is clear from these facts that chromosome binding by catechin, epicatechin and epigallocatechin must be dynamic and reversible to meet all functional and structural demands of the tissue.

## 5. Conclusion: Basic Principles of Flavanol Interactions in Nuclear Organization

1. Low molecular weight flavanols in the nuclei are controlled transcriptionally as shown by the homogeneinity of lineage cells (Figure 2);
2. The inherited pattern of nuclear flavanol expression is modified by epigenetic factors (Figure 4) as most dramatically shown during drought periods;
3. Thus, development and final differentiation of a cell is mirrored by changing subnuclear patterns of pale blue euchromatin, dark blue heterochromatin and different amounts of flavanols in the interchromosomal space (Figure 2);
4. All in all, the varying spatial densities of anchored or soluble flavanol molecules are expected to modify a series of physico-chemical parameters within the nucleosomal microenvironment (Figure 2).

## Figures and Tables

**Table 1:** Average values of the relative nuclear flavanol density (densitometric absorbance A 640) and the intranuclear variation (standard deviation, SD) as shown in the colored (Figure 1(a–y)).

Figures	Absorbance (640 nm)	Standard deviation SD	State	Relative SD	Relative SD
a1	21.6	4.6	active	21	16
a2	17.8	1.4	active	8	
b1	49.9	8.8	active	18	
b2	30.3	2.9	active	10	
c1	37.5	5.9	active	16	
c2	36.9	7.0	active	19	
d1	52.0	20.8	active	40	
n1	35.0	8.5	active	24	
w1	24.1	2.2	active	9	
x1	34.8	4.5	active	13	
x2	20.4	1.8	active	9	
y1	47.8	4.7	active	10	
h1	49.4	0.1	intermediate	0	11
i1	35.5	8.6	intermediate	24	
j1	82.7	7.9	intermediate	10	
k1	35.8	5.9	intermediate	16	
l1	53.7	6.6	intermediate	12	
m1	72.4	3.7	intermediate	5	
e1	96.0	8.6	silent	9	4
f1	31.3	1.0	silent	3	
g1	19.6	0.2	silent	1	
o1	49.4	4.0	silent	8	
p1	100.0	0.0	silent	0	
q1	magnification see r1	-	silent	-	
r1	45.7	3.3	silent	7	
s1	21.5	2.2	silent	10	
t1	0 --> heat	-	silent	-	
u1	88.4	0.5	silent	1	
v1	44.3	0.4	silent	1	

**Table 2:** Relative density of flavanols in nuclei. Densitometer absorbance at A 640 nm, mean values and intranuclear variation (SD, standard deviation); same lineages as in Table 2.

Lineage		1	2	3	4	5	6	7	8	1–8
**Silenced**	A	91	106	101	93	104	93	79	97	96
	SD	7.0	2.8	2.8	0.4	1.3	3.3	4.8	8.5	8.6
**Activated**	A	27	49	29	48	48	85	54	79	52
	SD	2.6	13.9	5.2	11.8	17.6	7.3	10.8	18.4	20.8

**Table 3:** Perimeter [μm] of cells (C), nuclei (N) and cell/nucleus ratio (R) of two cell lineages (silenced, (Figure 1(e)) and activated, (Figure 1(d))) with eight cells each.

Lineage		1	2	3	4	5	6	7	8	1–8
**Silenced**	C	51	49	70	54	53	66	45	60	56
	N	16	17	17	17	15	19	15	19	17
	R	3.2	2.9	4.1	3.2	3.5	3.5	3.0	3.2	3.3
**Activated**	C	40	50	35	37	41	39	48	45	42
	N	31	37	27	31	36	26	40	29	32
	R	1.3	1.4	1.3	1.2	1.1	1.5	1.2	1.6	1.3

**Table 4:** Phenolic compounds (mg/g DW) during microsporogenesis (pollen mother cells and tetrads) as compared with sprouting young needles.

	Catechin	Epigallocatechin	Epicatechin	Oligomers	Hydroxycinnamic acids	Flavonols
Microspores	8.6	0.6	7.1	3.3	2.9	0.06
Young needles	1.6	0.2	0.7	0.9	161.0	0.3

## Abbreviations

DMACA: p-dimethylaminocinnamaldehyde
